# Ambulatory arterial stiffness index among children and adolescents with chronic kidney disease a report from the Chronic Kidney Disease in Children Study

**DOI:** 10.21203/rs.3.rs-9349516/v1

**Published:** 2026-04-23

**Authors:** Susan M Halbach, Jennifer Roem, Michael F Schneider, Joshua Samuels, Susan L Furth, Bradley A Warady, Joseph T Flynn

**Affiliations:** University of Washington; Johns Hopkins Bloomberg School of Public Health: Johns Hopkins University Bloomberg School of Public Health; Johns Hopkins Bloomberg School of Public Health: Johns Hopkins University Bloomberg School of Public Health; The University of Texas Health Science Center at Houston; University of Pennsylvania Perelman School of Medicine; Children’s Mercy Hospital: Children’s Mercy Kansas City; University of Washington School of Medicine

## Abstract

**Background:**

To quantify the association between ambulatory arterial stiffness index (AASI) and hypertensive status, and to identify additional predictors of AASI, including body mass index (BMI), proteinuria, and mineral metabolism, in children and adolescents with chronic kidney disease (CKD).

**Methods:**

We analyzed data collected from 654 participants during 1283 study visits in the Chronic Kidney Disease in Children (CKiD) study. AASI was calculated from ambulatory blood pressure monitoring (ABPM) data as 1 minus the slope coefficient from a least squares linear regression of diastolic blood pressure (BP) on systolic BP. Hypertensive status was determined by clinic BP and ABPM.

**Results:**

At their first successful ABPM visit, the median age was 12 years, 61% were male, 15% had obesity and the median eGFR was 49 ml/min/1.73m^2^. Over half (57%) reported current antihypertensive therapy use with RAAS inhibitors, yet 42% met criteria for abnormal ABPM (elevated mean total/awake/sleep DBP/SBP per updated guidelines). Masked hypertension (MH) was common (27%) and associated with significantly higher AASI compared to being normotensive (difference in mean AASI: +0.051, 95% CI: 0.032, 0.071). Male sex and higher BMI were associated with higher AASI, but the presence of proteinuria, abnormal serum calcium or phosphate were not.

**Conclusion:**

AASI is associated with MH and BMI in children and adolescents with CKD and may be a useful non-invasive measure of vascular stiffness in this population.

## Introduction

Young adults with childhood-onset chronic kidney disease (CKD) have significantly elevated risk of cardiovascular mortality compared to the general population [1]. The pathophysiology leading to premature cardiovascular disease in those with childhood-onset CKD is likely multifactorial and not completely understood. One possible pathway is early vascular aging (EVA), whereby abnormal endothelial function and increased arterial stiffness develop at an accelerated rate [2–4]. Among adults, increased arterial stiffness is associated with major cardiovascular events as well as diastolic dysfunction, cognitive impairment, reduced glomerular filtration rate (GFR) and a faster rate of GFR decline [5–11].

Pulse wave velocity (PWV) is the most common non-invasive method to measure vascular stiffness, but its use remains confined to the research setting and requires trained staff to ensure accuracy and reproducibility [12]. Twenty-four-hour ambulatory blood pressure monitoring (ABPM) is used commonly in the management of children with CKD, and from the blood pressure readings obtained during ABPM, it is possible to calculate the ambulatory arterial stiffness index (AASI), which has been proposed as a surrogate marker of vascular stiffness [13]. Studies in adults have demonstrated a positive correlation between AASI and other markers of vascular stiffness and have established AASI as an independent predictor of cardiovascular events, particularly stroke [14, 15]. There are fewer studies in children, but data suggest an association between AASI and metabolic syndrome, obesity and hypertension in otherwise healthy children and those with type 1 diabetes mellitus [16–18]. However, despite the high cardiovascular risk in children and adolescents with CKD, AASI data in this population are limited.

The primary objective of this study was to quantify the relationship between AASI and markers of cardiovascular disease in children and adolescents with CKD. Specifically, we hypothesized that AASI would be positively correlated with hypertensive phenotype, body mass index (BMI) and severity of CKD. A secondary aim was to describe changes in AASI with concurrent changes in ABPM, with the hypothesis that ABPM changes consistent with poorly controlled or developing hypertension would be associated with concomitant increases in AASI.

## Methods

### Study Population

We used data from the Chronic Kidney Disease in Children (CKiD) study, a multicenter cohort study of children, adolescents and young adults with mild to moderate CKD (median baseline estimated glomerular filtration rate (eGFR) of 53 ml/min/1.73m^2^) from more than 50 centers across North America. Additional details on the CKiD study design and inclusion criteria have been previously published [19]. The study design and conduct were approved by an observational study monitoring board appointed by the National Institute of Diabetes and Digestive and Kidney Diseases and by central and local institutional review boards of the participating centers. Written informed consent/assent was obtained from participants and their families. All annual participant-visits with successful ABPM and casual blood pressure (BP) data were eligible for inclusion in our analyses.

### Blood Pressure Measurements

Casual BP measurements were obtained at all annual study visits. All casual BP measurements were obtained by auscultation using aneroid sphygmomanometers (Mabis MedicKit 5, Mabis Healthcare, Waukegan, IL) provided by the CKiD Clinical Coordinating Centers. Prior to BP measurement, trained study staff measured the child’s mid-arm circumference and selected the appropriate cuff size. The child remained at rest for at least 5 minutes before cuff inflation and three measurements were obtained, with 30 seconds between each measurement. The recorded BP for the study visit was the average of the three auscultated measurements [20].

ABPM was conducted one year after the baseline visit (i.e., at the second annual visit) and every two years thereafter (i.e., at every other annual visit) using the Spacelabs 90217 monitor (SpaceLabs Healthcare, Issaquah, WA). All ABPMs were placed on the participant at the home institution, but data were sent to a single center for analysis. All study participants were provided with a patient diary to record sleep and wake times as well as medication administration times or unusual activities. Monitors were programmed to obtain BP readings every 20 minutes throughout the 24-hour period. Once the monitor was returned and data were downloaded, each ABPM was assessed for adequacy and participants were offered a second recording in cases of inadequate readings. Monitors worn for ≥ 24 hours with ≥ 18 hours with ≥ 1 reading per hour were considered adequate for interpretation. Additionally, a balance of readings during both the sleep and wake periods was required (≥ 1 BP per hour in ≥ 75% of each of the two periods). These criteria are consistent with prior CKiD analyses using ABPM [21].

AASI was calculated from each adequate ABPM recording by the CKiD ABPM coordinating center as 1 minus the slope coefficient from a least squares linear regression of diastolic BP on systolic BP [13, 14].

### Primary Exposure

Hypertensive status was defined using both casual and ABPM data, based on current American Academy of Pediatrics (AAP) and American Heart Association (AHA) clinical guidelines [22, 23]. Casual BP was classified as normal, elevated, stage 1 hypertension, or stage 2 hypertension. Participants met criteria for abnormal ABPM if the mean total, wake, and/or sleep SBP or DBP was ≥ 95th sex-height-specific percentile or ≥ adult ABPM cutoff values (125/75, 130/80, 110/65 for total, wake, and sleep SBP/DBP, respectively) when participants were < 13 years old and if the mean total, wake, and/or sleep SBP or DBP was ≥ adult ABPM cutoff values for hypertension when participants were ≥ 13 years old [22, 24]. Using this information along with casual BP obtained at the same study visit, participants were classified into one of the following four categories: ambulatory hypertension (casual BP stage 1–2 and abnormal ABPM), white-coat hypertension (casual BP stage 1–2 and normal ABPM), masked hypertension (MH; casual BP in normal or elevated range and abnormal ABPM) and normal (casual BP in normal or elevated range and normal ABPM). Data on anti-hypertensive medication use were collected and analyzed, but we did not consider medication use in defining hypertensive status.

### Additional Covariates

Other covariates were measured at baseline and at follow-up visits concurrent with AASI determinations. Demographic characteristics included biological sex and age. Age-sex-specific height z-scores were determined based on the 2000 Centers for Disease Control (CDC) growth charts [25]. We examined CKD etiology (characterized as glomerular and non-glomerular), as well as urine protein to creatinine (UP/C) ratio (characterized as nephrotic range if UP/C > 2 mg/mg) and eGFR [26]. Laboratory measures included serum calcium and phosphate, each classified as low, normal and high based on age-specific thresholds [27]. Blood and urine samples for laboratory data were collected at the same visit as the casual BP and ABPM and were analyzed in the central study lab (University of Minnesota, Minneapolis MN). Participant demographic and medical history information, including medication administration, were gathered by self-report and parent report at each study visit.

### Statistical Analysis

Box-percentile plots showed the distribution of AASI by hypertensive status. Univariate and multivariable linear regression quantified the relationship AASI had with hypertensive status and each of the covariates. These linear regression models used generalized estimating equations (GEE) to account for repeated measurements of AASI within the same individuals across follow-up visits. Additionally, box-percentile plots summarized the annualized change in AASI between two consecutive ABPM visits, where time between visits was < 2.5 years, overall and by concurrent transition in ABPM status (i.e., remained normal between two visits, became normal, became abnormal, and remained abnormal). All analyses were done using R version 3.6.1. Regression models with GEE were implemented using the R package “geepack.”

## Results

Visits from the CKiD cohort (6275 visits from 1100 participants) ineligible for analysis included: 4529 without ABPM, 224 with unsuccessful ABPM, 40 with missing hypertensive status, and 199 with incomplete data on covariates of interest. Our final study population and analyses included 1283 person-visits from 654 children with complete data on all covariates of interest. Participants contributed an average of two person-visits (range: 1 to 6 visits) of data to the analyses. Characteristics of the population at the first successful ABPM visit are described in Table 1. Most of the participants were male (61%), the median age was approximately 12 years, and the median eGFR was 49 ml/min|1.73m^2^, with 95% having an eGFR between 15 and <90 ml/min/1.73m^2^. Abnormal ambulatory blood pressure was observed in 276 participants, of whom 65% (n = 179) were classified as having MH and 35% (n = 97) ambulatory hypertension. Use of ACEi/ARB antihypertensive medications was common (57%). Sixty-nine percent of participants had a BMI classified as normal, but a substantial number of participants had overweight (12%) or obesity (15%). Among all 1283 person-visits, 347 (27%) were classified as having MH, with 36% (n=126) meeting that criteria by demonstrating wake and sleep MH, 53% (n=185) with isolated nocturnal MH and 11% (n=36) with isolated daytime MH.

**Figure 1:**
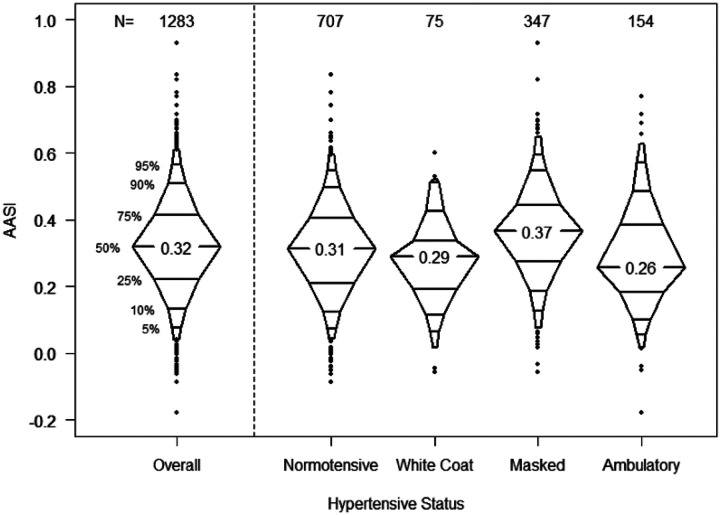
Distribution of measured AASI by hypertensive status (654 participants contributing a total of 1283 person-visits).

The distribution of AASI by hypertensive status is shown in Figure 1 for all 1283 study visits. The median AASI for all study participants was 0.32 (inter-quartile range (IQR): 0.22–0.41); those with MH had the highest median AASI (0.37, IQR: 0.27 – 0.44), while those with ambulatory hypertension had the lowest median AASI (0.26, IQR: 0.18 – 0.38). When analyzed dichotomously as normal or abnormal ABPM, those with abnormal ABPM had higher AASI compared to those with a normal ABPM (0.34 vs. 0.31, Supplemental Figure 1). Median AASI also differed by whether participants were hypertensive during the day (0.25), during sleep (0.37) or both (0.32).

Table 2 presents results from the unadjusted and adjusted models of AASI. In the unadjusted models, those with white coat and ambulatory hypertension had lower mean AASI (−0.035 [95% CI: −0.066, −0.004] and −0.026 [95% CI: −0.055, +0.003], respectively) than those who were normotensive. Those with MH had a significantly higher mean AASI (+0.051 [95% CI: +0.032, +0.071]). Other variables associated with AASI were older age, male sex, greater height z-score, and ACEi/ARB use. A BMI classification of overweight or obese was associated with a higher AASI. After adjustment, those with white coat and ambulatory hypertension still had lower mean levels of AASI (−0.036 [95% CI: −0.067, −0.005] and −0.033 [95% CI: −0.062, −0.003], respectively), while those with MH had higher AASI (+0.047 [95% CI: +0.028, +0.066]) compared to those who were normotensive as well as those with ambulatory hypertension and white-coat hypertension. eGFR, CKD etiology (glomerular vs. non-glomerular) and proteinuria were not associated with AASI. Similarly, abnormal serum calcium and phosphorus (high or low) were not associated with AASI. There was no significant relationship between carotid intima medial thickness (cIMT) and AASI (data not shown).

**Figure 2:**
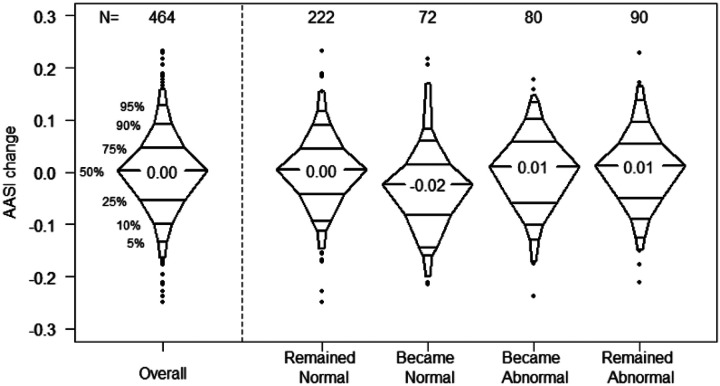
Annualized change in AASI change by transitions in ABPM status, 292 participants contributing a total of 464 visit-pairs with <2.5 years between visits

Figure 2 depicts the annualized change in AASI between visits both overall and by change in ABPM status between visits. The median annualized change in AASI was +0.003 per year across 464 visit-pairs from 294 participants. Of those 464 visit-pairs, 48% (n = 222) had the participant remaining normal, in 16% (n = 72) the participant became normal, in 17% (n = 80) the participant became abnormal, and in 19% (n = 90) the participant remained abnormal. A negative median annualized AASI change (−0.02) was seen in children who previously had abnormal ABPM and had a normal ABPM at their subsequent visit. Those who remained normal, remained abnormal, or became abnormal between consecutive visits all saw minimally positive median annualized AASI changes (+0.005, +0.011, + 0.013, respectively).

## Discussion

The main findings of our study are that among BP phenotypes in children with CKD, compared to normotension, MH was significantly associated with an increased AASI. In addition, we found a positive dose-response relationship between BMI category and AASI. The association between increased AASI and hypertension has been shown in other pediatric populations and recently in a pediatric CKD cohort study in Taiwan [28]. Sulakova et al. found an increased AASI among diabetic children with both white-coat hypertension and sustained hypertension compared to diabetic normotensive children and non-diabetic normotensive controls [16]. A study comparing children with both primary and secondary hypertension found not only increased AASI among hypertensive children compared to healthy controls, but a dose-response relationship between duration of hypertension and AASI [29]. Hsu et al. found that, among children with CKD, those with mean 24-hour, awake, and sleeping BPs > 95th percentile had significantly higher AASI [30]. Using a broad definition for hypertension based on ABPM findings (elevated mean sleep/wake, SBP/DBP), our finding that a higher AASI is associated with MH is consistent with these prior studies.

Most of the children in the CKiD study meeting criteria for hypertension based on 24-hour ABPM [22] had MH (69%), with a smaller portion having ambulatory hypertension (31%). Among abnormal ABPM recordings, only MH was significantly associated with an increased AASI compared to normotension. The reason for this is unclear, but it could be related to the higher proportion of children in the MH group having nocturnal hypertension. This is supported by the finding that participants in our study with isolated nocturnal hypertension had the highest mean AASI among all groups. Although the pathophysiology of the development of vascular stiffness is not fully understood, one hypothesis is that vascular changes and stiffness precede the development of hypertension. Among children with primary hypertension, markers of increased vascular stiffness are often present when hypertension is diagnosed, suggesting a complex process of early vascular aging [31]. Other studies have compared AASI between groups based on normal/abnormal ABPM and it remains unclear which abnormal patterns may be the most predictive. Given that a large proportion of CKD children with MH had nocturnal hypertension (with approximately half having isolated nocturnal hypertension), future studies looking at the contribution of abnormal diurnal BP patterns to the development of cardiovascular disease are important.

Our finding that the median change in AASI was negative among those who went from an abnormal first ABPM to a normal second ABPM suggests that correctly identifying and adequately controlling BP could potentially impact the progression of vascular stiffness among children with CKD. Although the differences were small and most study participants had only two ABPM studies available for comparison, this finding could represent an opportunity for interventions to mitigate cardiovascular risk among youth with CKD, particularly given the high proportion of hypertensive children with MH. Studies in adults indicate that not only is MH more prevalent in patients with CKD, but it is associated with increased cardiovascular morbidity and mortality, including markers of vascular stiffness [32]. Additionally, a recent large population study showed that the addition of AASI to traditional risk factors improved model prediction for cardiovascular events and mortality among adults, demonstrating potential clinical utility for risk stratification [33]. The high proportion of children with MH in the CKiD cohort is an important finding and presents an opportunity for intervention.

Our study findings are also consistent with prior studies demonstrating a relationship between obesity and increased AASI. Obesity and BMI appear to be independent predictors of arterial stiffness even when controlling for hypertension, which is similar to studies demonstrating obesity as an independent predictor of left ventricular hypertrophy regardless of hypertensive status [17, 34]. Other factors thought to contribute to the development of vascular stiffness and vasculopathy among individuals with CKD, such as serum calcium and serum phosphorus, were not associated with AASI. These markers may not be representative surrogate markers for developing vascular stiffness or the measurement range may be too narrow to detect meaningful differences.

The lack of association between eGFR and AASI is consistent with the relationship found between other intermediate markers of cardiovascular disease and eGFR in children, specifically carotid intima media thickness (cIMT) [35]. We also did not see a relationship between markers of mineral metabolism (serum calcium, serum phosphorus) and AASI. Despite the knowledge that vascular calcification occurs at higher rates in individuals with CKD, the pathway for mineral metabolism is complex and serum levels of calcium and phosphorus alone may not be the only predictors. At least one prior study has found serum phosphorus to be a predictor of a composite cardiovascular score (containing left ventricular mass index, cIMT and central pulse wave velocity) [36].

There are several limitations with our study. As mentioned, the spectrum of CKD severity is relatively narrow in the CKiD study, which may not allow us to detect meaningful differences in AASI as CKD progresses. Secondly, ABPM data were only available for an average of two study visits per participant spanning a 2-year timeframe, which may not be sufficiently long enough to detect meaningful changes in arterial stiffness. Although we included the use of ACEi/ARB as a covariate, the specific association between class of antihypertensive and AASI was not assessed in our analysis, as there are likely confounders, such as different indications for taking some of these medications and varying practice patterns. Lastly, we did not have non-CKD controls for comparison. Although we

Although there is no accepted “normal” AASI value, the mean AASI reported in healthy controls for most studies in children ranges from 0.19–0.23, which is lower than the median (0.31) value of the normotensive group in our CKD study [16, 17]. Additionally, the study by Hsu et al. found a mean AASI of 0.36 in their entire study cohort, in which over 50% of the study subjects had CKD Stage 1 [28, 30]. This at least suggests that children with CKD may have higher AASI and, potentially, increased vascular stiffness even early in the disease process.

Despite these limitations, our study has important implications for the assessment of overall cardiovascular risk in children with CKD. Early vascular aging may be an important component in the development of cardiovascular disease among young adults with pediatric-onset CKD, which is the leading cause of mortality in this population. In turn, our findings support current clinical guidelines which recommend routine performance of ABPM in high-risk populations, including children with CKD and normal clinic blood pressure readings [21, 22]. The ready availability of AASI from ABPM provides an additional tool for providers to gauge overall cardiovascular risk when screening for hypertension in children with CKD and can be used to guide treatment and frequency of follow up. Additional longitudinal data in children with primary hypertension and CKD will be helpful in further characterizing the development of cardiovascular disease in this high-risk group and the contribution of early vascular aging. Comparisons of AASI between children with CKD and age-matched controls would also be useful in understanding how these measures differ between the two populations.

## Supplementary Material

Supplementary Files

This is a list of supplementary files associated with this preprint. Click to download.

- SupplementalFigure1.docx
- Halbachgraphicabstract2026.pptx
- STROBEchecklistcohortHalbach.docx
- SupplementalTable1.docx

## Figures and Tables

**Table 1.** Study population characteristics at first successful ABPM visit, N=654.

Characteristics	Median [interquartile range] or % (n).
Male sex	61% (398)
Age, years	12.4 [9.2, 15.9]
CKD duration, years	9.7 [6.4, 13.9]
Black race	19% (121)
Age-sex-specific height z-score	−0.5 [−1.2, 0.3]
BMI	
Underweight (<5^th^ percentile)	4% (25)
Normal (5^th^ to <85^th^ percentile)	69% (448)
Overweight (85^th^ to <95^th^ percentile)	12% (82)
Obese (≥95^th^ percentile)	15% (99)
Glomerular CKD	26% (168)
Urine protein/creatinine > 2 (mg/mg)	10% (65)
eGFR (ml/min per 1.73m^2^)	49.3 [35.1, 63.6]
CKD stage	
1 (eGFR ≥ 90)	4% (30)
2 (60 ≤ eGFR <90)	27% (178)
3 (30 ≤ eGFR <60)	51% (331)
4 (15 ≤ eGFR <30)	17% (109)
5 (eGFR <15)	1% (6)
Calcium‡	
Low	16% (105)
Normal	82% (533)
High	2% (16)
Phosphate‡	
Low	3% (21)
Normal	80% (521)
High	17% (112)
Currently taking ACEi/ARB	57% (370)
Clinical hypertension (Stage 1 or 2)	21% (137)
ABPM hypertension (abnormal ABPM)	42% (276)
Hypertensive status	
Normotensive	52% (338)
White coat	6% (40)
Masked	27% (179)
Ambulatory	15% (97)

‡ Based on age-specific threshold (ref 27)

**Table 2.** Predictors of ambulatory arterial stiffness index (AASI), N=654 participants contributing a total of 1283 person-visits.

Characteristics	Unadjusted Analyses Estimate± (95% CI)	Adjusted Analysis Estimate±† (95% CI)
Hypertensive status		
Normotensive	ref	ref
White Coat	**0.035 (−0.066, −0.004)**	**0.036 (−0.067, −0.005)**
Masked	**0.051 (0.032, 0.070)**	**0.047 (0.028, 0.066)**
Ambulatory	−0.026 (−0.055, 0.003)	**0.033 (−0.062, −0.003)**
Male sex	**0.050 (0.030, 0.069)**	**0.051 (0.031, 0.070)**
Age, per year	**0.003 (0.001, 0.005)**	0.002 (−0.001, 0.005)
Age-sex-specific height z-sore, per SD	0.009 (−0.0003, 0.017)	0.002 (−0.006, 0.010)
Body Mass Index		
Underweight (<5^th^ percentile)	−0.024 (−0.074, 0.027)	−0.034 (−0.082, 0.013)
Normal (5^th^ to <85^th^ percentile)	ref	ref
Overweight (85^th^ to <95th percentile)	**0.044 (0.017, 0.070)**	**0.039 (0.014, 0.063)**
Obese (≥95^th^ percentile)	**0.067 (0.040, 0.094)**	**0.062 (0.035, 0.090)**
Glomerular CKD diagnosis	0.010 (−0.015, 0.035)	0.002 (−0.024, 0.027)
Urine protein/creatinine > 2 (mg/mg)	0.016 (−0.013, 0.046)	0.017 (−0.014, 0.048)
eGFR, per 10 ml/min|1.73m^2^ decrease	−0.0001 (−0.005, 0.005)	−0.003 (−0.008, 0.002)
Calcium‡		
Low	−0.001 (−0.025, 0.024)	−0.002 (−0.028, 0.023)
Normal	ref	ref
High	0.004 (−0.047, 0.054)	0.007 (−0.045, 0.058)
Phosphate‡		
Low	−0.009 (−0.059, 0.041)	0.014 (−0.036, 0.064)
Normal	ref	ref
High	0.007 (−0.013, 0.027)	−0.011 (−0.031, 0.010)
Currently taking ACEi/ARB	**0.025 (0.007, 0.044)**	**0.020 (0.002, 0.039)**

± Estimates are based on a linear regression model using generalized estimating equations to account for repeated measurements within participants during study follow-up.

† The adjusted analysis includes all covariates listed.

‡ Based on age-specific threshold (ref 27)
